# Shared Visual Attention and Memory Systems in the *Drosophila* Brain

**DOI:** 10.1371/journal.pone.0005989

**Published:** 2009-06-19

**Authors:** Bruno van Swinderen, Amber McCartney, Sarah Kauffman, Kris Flores, Kunal Agrawal, Jenée Wagner, Angelique Paulk

**Affiliations:** 1 Queensland Brain Institute, The University of Queensland, Brisbane, Queensland, Australia; 2 The Neurosciences Institute, San Diego, California, United States of America; Max-Planck-Institut fuer Neurobiologie, Germany

## Abstract

**Background:**

Selective attention and memory seem to be related in human experience. This appears to be the case as well in simple model organisms such as the fly *Drosophila melanogaster*. Mutations affecting olfactory and visual memory formation in *Drosophila*, such as in *dunce* and *rutabaga*, also affect short-term visual processes relevant to selective attention. In particular, increased optomotor responsiveness appears to be predictive of visual attention defects in these mutants.

**Methodology/Principal Findings:**

To further explore the possible overlap between memory and visual attention systems in the fly brain, we screened a panel of 36 olfactory long term memory (LTM) mutants for visual attention-like defects using an optomotor maze paradigm. Three of these mutants yielded high *dunce*-like optomotor responsiveness. We characterized these three strains by examining their visual distraction in the maze, their visual learning capabilities, and their brain activity responses to visual novelty. We found that one of these mutants, *D0067*, was almost completely identical to *dunce^1^* for all measures, while another, *D0264*, was more like wild type. Exploiting the fact that the LTM mutants are also Gal4 enhancer traps, we explored the sufficiency for the cells subserved by these elements to rescue *dunce* attention defects and found overlap at the level of the mushroom bodies. Finally, we demonstrate that control of synaptic function in these Gal4 expressing cells specifically modulates a 20–30 Hz local field potential associated with attention-like effects in the fly brain.

**Conclusions/Significance:**

Our study uncovers genetic and neuroanatomical systems in the fly brain affecting both visual attention and odor memory phenotypes. A common component to these systems appears to be the mushroom bodies, brain structures which have been traditionally associated with odor learning but which we propose might be also involved in generating oscillatory brain activity required for attention-like processes in the fly brain.

## Introduction

We have previously shown that attention-like processes can be studied in *Drosophila* using both electrophysiological and behavioral approaches [1], [2], and that mutations in learning and memory pathways modulate attention-like responses in flies [3]. Mutations in *dunce* (a cyclic AMP phosphodiesterase) and *rutabaga* (an adenylyl cyclase), which compromise memory formation via the cyclic AMP pathway [4], also produce defects in short-term processes relevant to selective attention. These defects include decreased responsiveness to visual novelty and decreased distractibility by competing visual stimuli. Interestingly, such attention defects were associated with *increased* behavioral responsiveness to moving gratings in an optomotor choice maze, where *dunce* and *rutabaga* mutants performed twice as strongly as wild-type flies. Why should learning and memory mutants display such strong visual reflexes?

Our interpretation of the stronger performance of these mutants in the optomotor choice maze is that these animals are less able to suppress prepotent, or reflexive, responses (to the moving grating in the maze). Thus, their attention-like defects (as assessed electrophysiologically [3]) are manifested behaviorally by a reduced ability to suppress a reflex such as the optomotor response. We suggested that such defective suppression abilities were a possible source of *dunce* mutant's learning deficits in olfactory [5] and/or visual [6] assays, where ongoing stimulus suppression dynamics may play a role in the normal acquisition or retrieval of memories. The reduced ability of *dunce* mutants to suppress prepotent reflexes is also evident in another visual paradigm, aversive phototaxic suppression, where wild-type flies learn to avoid a lit chamber associated with quinine whereas *dunce* fail to suppress their phototaxic reflex over time [7].

We therefore used optomotor responsiveness in the maze as a means of uncovering other pathways that might compromise visual attention in flies, with the prediction that strong optomotor reflexes in other mutants might be associated with distinct visual attention defects. Rather than performing a large-scale blind screen, we decided to focus on a panel of mutant strains found to be defective in olfactory long term memory (LTM) [8]. Our focus on LTM or plasticity genes stems from an earlier result showing that *dunce* attention defects could only be rescued by expression of the wild-type protein throughout development [3]. We hypothesize that LTM genes represent possible downstream targets of cyclic AMP signaling (i.e., in the same cells), and thus might be involved in establishing the neuronal architecture required for attention-like processes in the adult during brain development. We predicted that, like *rutabaga^2080^* and *dunce^1^*, some of these olfactory LTM mutants might also be characterized by distinct attention-like defects in our behavioral and electrophysiological paradigms because LTM pathways are likely to modulate attention. Crucially, the genetic lesions in many of these LTM strains are caused by Gal4 constructs stably inserted into the genome [8]. These elements therefore also function as enhancer traps to allow for a complete examination of the neural architecture possibly compromised by the mutations. By examining the neuroanatomy, we could therefore uncover possible common substrates of visual attention and olfactory memory in strains where both phenotypes are affected. The Gal4 elements would then also serve as molecular ‘handles’ for active manipulation of circuits affecting attention and memory [9].

Among 36 LTM mutants, we found three strains with optomotor responsiveness as high as *dunce* mutants, namely *D0264*, *D0067*, and *D0177* (named Rafael, Norka, and Toi, respectively, in the publication where they were first described [8]). Although these three strains produced similar levels of high optomotor responsiveness, they displayed strikingly different characteristics for attention-like processes, with *D0067* most similar to *dunce* in both behavioral and electrophysiological paradigms. We also found all three strains to be defective in some aspects of visual learning. By using the inserted elements as Gal4 complements to either rescue *dunce* defects or silence synaptic activity, we found that central brain structures, namely the mushroom bodies, may be key to modulating visual attention phenotypes in *Drosophila*.

## Results

### Optomotor Behavior

**Figure 1:**
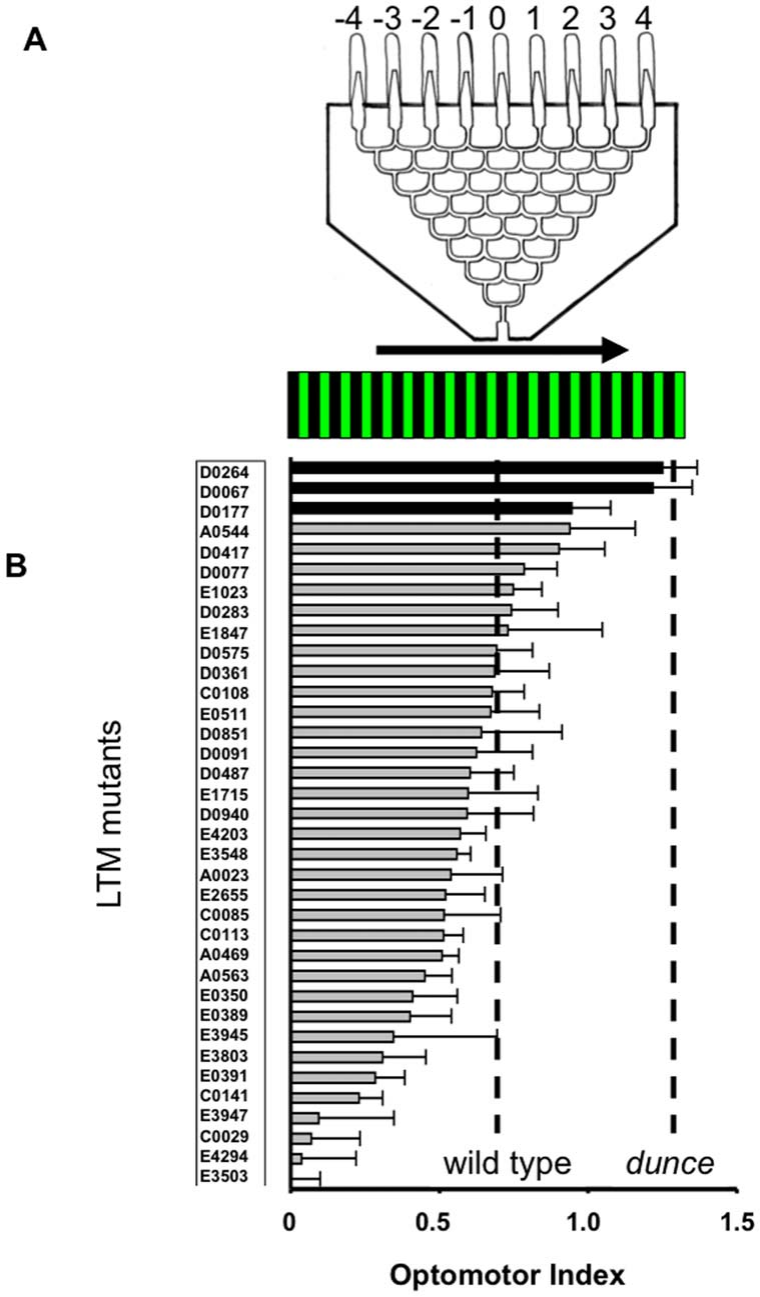
The optomotor maze paradigm. A. The eight-level choice maze. Arrow: grating direction. The distribution of flies among the nine end tubes determines a strain's optomotor response. B. Optomotor Index (±s.e.m., see Methods) for 36 long term memory (LTM) mutants in response to a green and black moving grating. The average response levels for *dunce* mutants and wild type are indicated by dashed lines. Significant responses for LTM variants (darkened histograms) were determined by *t-*test (*P<*0.05) against wild type.

Mutations in the memory genes *dunce* and *rutabaga* increase visual responsiveness to moving gratings in an optomotor maze [3]. We hypothesized that defective attention-like processes in other genes associated with memory formation might also be uncovered by examining the behavior of other olfactory memory mutants in the optomotor maze. We therefore tested 36 long-term memory (LTM) mutants for visual responsiveness to moving gratings in our optomotor maze paradigm (Figure 1A). These mutant strains were first isolated in a screen for defective LTM in an olfactory paradigm [8]. As shown in Figure 1B, the 36 mutants display a wide range of optomotor responsiveness levels, from zero responsiveness to very strong optomotor responses (as observed in *dunce* and *rutabaga* mutants) [3]. We focused on three olfactory LTM variants displaying significantly greater optomotor responsiveness than wild-type flies: *D0264*, *D0067*, and *D0177* (Figure 1B, black histogram bars). By selecting LTM mutants with *dunce*-like responsiveness in the optomotor maze, we attempted to uncover overlapping systems in the brain relevant to both memory formation and attention. Our investigation of these selected LTM variants progressed as follows: visual distraction effects in the maze, visual learning behavior, and neural correlates of visual attention. We then use the elements as Gal4 drivers to investigate their capacity for rescuing *dunce* attention defects and for active control of these neurons to modulate attention-like phenotypes. Our results are thus divided in two broad sections: first, an analysis of the LTM mutants for visual attention and memory phenotypes, and second an analysis of possible Gal4 networks affecting visual attention in flies.

**Figure 2:**
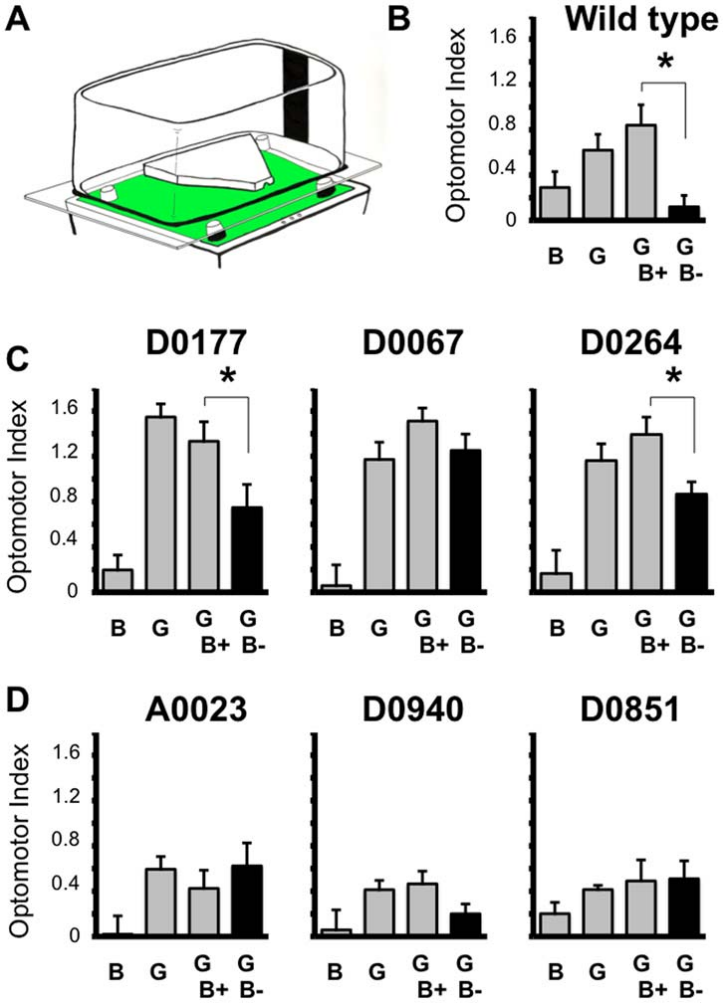
Distraction paradigm. A. Distractibility (see Methods) is demonstrated by significantly reduced (*, *P*<0.05) optomotor responsiveness in the presence of a competing static black bar on the side opposite of optomotor flow compared to the image being on the same side [1]. B. Data for wild-type flies. B, response (±s.e.m.) to the static bar alone; G, response to the moving grating alone; GB+, response when the bar is on the same side as the direction of the moving grating; GB-, response when the bar is on the side opposite to the direction of grating movement. The bar on the side opposite to grating movement (black histogram) significantly reduces the optomotor response. C. Distractibility profile for the three strongest optomotor performers, *D0177*, *D0067*, and *D0264*. D. Distractibility profile for three average optomotor performers, *A0023*, *D0940*, and *D0851*.

To examine how attention-like processes might vary across the LTM mutants, we first tested the distractibility of the flies. Flies progressing through the optomotor maze can see objects surrounding the maze, and the position of these objects influences their optomotor response [1] (Figure 2A). For example, for wild-type flies, a black bar positioned on the opposite side of optomotor flow abolishes the flies' response to the moving grating, and this effect has been invoked as a measure of distractibility [1], [3](GB- in Figure 2B). *dunce* mutants are unaffected by such static distracters, suggesting a defect in attention in these flies [3]. *D0067* and *D0264*, which responded as strongly as *dunce* in the optomotor maze, displayed different behaviors when presented with distracters. Whereas *D0067* was similar to *dunce^1^* in not being distracted by the competing visual stimulus, *D0264*, also a strong responder to the optomotor stimulus, was somewhat distractible, though not as strongly as wild type (a 37% attenuation versus 78% in wild type) (Figure 2C). Optomotor responses in *D0177* were also significantly decreased when the distracter was present (44% decrease, Figure 2C). Differences among these strains suggest that strong optomotor performance does not necessarily imply defects in perceiving static visual stimuli in competition with a moving grating. To compare this distraction phenotype in other LTM mutants, we randomly chose and tested three strains with wild-type optomotor response levels, namely *A0023*, *D0940*, and *D0851* (see Figure 1B). We found that two of these LTM strains with normal optomotor responsiveness were also unaffected by the visual distracter, like *dunce^1^* and *D0067* (Figure 2D). This further supports our observation that optomotor performance and visual distraction effects are separable.

### Visual Learning

Even though *D0264*, *D0067*, and *D0177* were isolated for defective LTM in an olfactory paradigm, they display distinct behaviors in the optomotor maze, suggesting broader defects in short-term behavioral processes. These strains have never been tested for visual learning paradigms, partially because the best visual learning paradigms for *Drosophila* are single animal assays requiring flight (the advantages and disadvantages of the tethered flight paradigm are reviewed in [10]). We tested these strains for visual learning using a modified version of a walking paradigm that pairs visual stimuli (the conditioned stimuli) with shaking (the unconditioned stimulus, see Methods and Supporting Information Movie S1). Wild-type flies learn to avoid visuals associated with shaking, whereas *dunce^1^* fails to show learning [11]; Figure S1). Our three high optomotor performers (*D0264*, *D0067*, and *D0177*) all failed to show learning in this visual paradigm (Figure S1). The fact that olfactory learning is normal in these mutants (only long-term memory is compromised) [8] highlights a difference between visual and olfactory paradigms with regard to classical conditioning.

The above learning paradigm reveals a performance index after training (see Methods), and thus may not best uncover subtle differences among mutants. Indeed, all three olfactory LTM mutants failed to learn, although differences in performance among these strains were evident during training (Figure S1). Furthermore, different sensitivities to the unconditioned stimulus (shaking) might also influence the learning phenotype, as might other aversive cues accumulating in the tubes during training, such as CO_2_ [12]. To further address visual learning through examining sensitization, we modified our optomotor maze paradigm: we ran flies through the maze multiple times to see if responses were affected by repeated exposure to the maze and visuals (see Methods). Interestingly, wild-type flies displayed stronger optomotor responses during repeated maze runs when they were performed in immediate succession (Figure S2), resulting in optomotor scores as high as *dunce^1^* mutants by the third exposure to the optomotor maze. Re-running *dunce* flies in the maze slightly increased responses though the difference was not significant (1.63±0.29 versus 1.27±0.17 for baseline). With a strong performer such as *dunce^1^* one might suspect a ceiling effect for the behavior, where even higher responses might be unlikely in this 8-level choice design. Instead, we found that *D0264* optomotor performance (which is already as high as *dunce* at baseline) was significantly increased by the third exposure to the maze, to 2.3±0.13. Neither *D0067* nor *D0177* displayed any such significant increases in optomotor responsiveness following repeated exposure (Figure S2). These experiments revealed two valuable points: first, some mutants perform even better than *dunce^1^* in the maze under certain “learning” conditions (there is no ceiling at an optomotor score of ∼1.2), and second, a strong optomotor performance does not always imply a lack of plasticity. *D0264*, with a strong optomotor response, can improve its performance with repeated exposure. Interestingly, *D0067*, which was not distracted by a competing visual object (Figure 2C) also showed no sensitization in the maze, making it most like *dunce^1^*.

Taken together, our behavioral experiments show that three olfactory LTM mutants, as well as *dunce^1^*, are significantly different than wild type in a variety of visual paradigms. This is consistent with the mutants having more generalized “cognitive” defects, which we now investigate further using electrophysiology.

### Neural correlates

**Figure 3:**
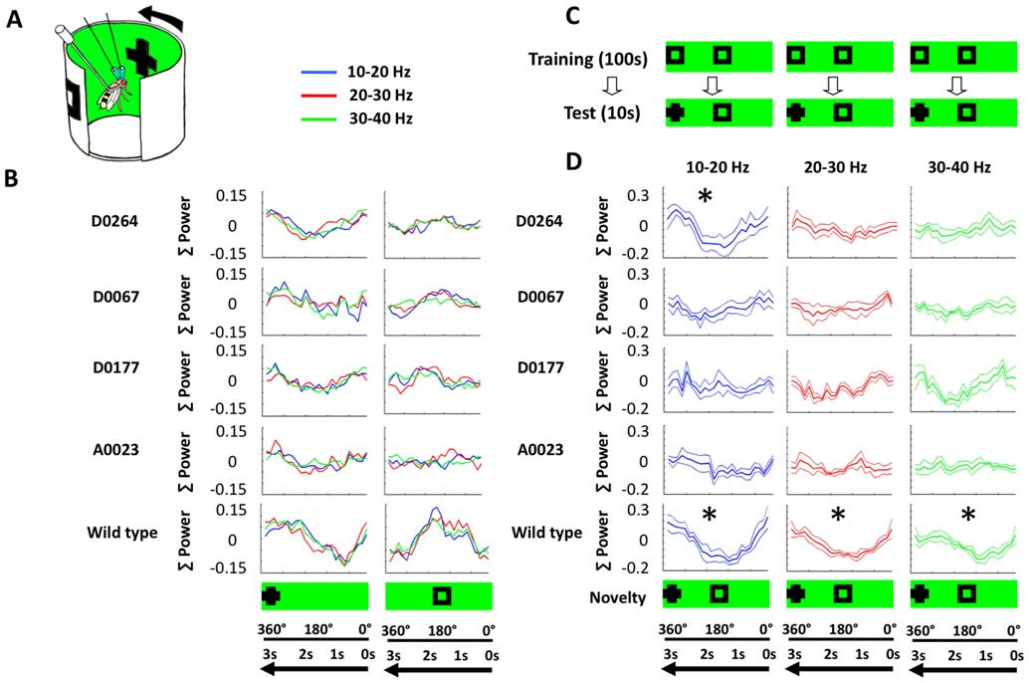
Brain responses. A. Recording arena. The tethered fly is implanted with two recording electrodes and placed in the center of an LED arena on which a moving visual panorama can be displayed. Arrow: visual panorama rotation. B. Baseline responses to the visual objects presented alone, for the 3 top optomotor performers (*D0264* (N = 6), *D0067* (N = 6), and *D0177* (N = 5), for an average optomotor performer, *A0023* (N = 4), and for wild type (N = 4). Average responses in three frequency ranges (10–20 Hz, blue; 20–30 Hz, red; 30–40 Hz, green) are shown. The matching position of either object (cross or square) is indicated in the schema at the bottom. Σ Power is the zero-meaned, normalized power, summed within each frequency domain and averaged among individuals within a genotype. C. Novelty paradigm. Flies are exposed to two identical squares (in black, depicted on the unwrapped green panorama) for 100 s (Training), immediately followed by presentation of a cross and a square at the same positions (Test). This is repeated 5 times to generate averaged responses for the 10 s following a novelty transition. D. Brain responses to novelty. Novelty responses (±s.e.m.) in four LTM mutants across three LFP frequency domains (blue: 10–20 Hz; red: 20–30 Hz; green: 30–40 Hz). The matching position of either object (cross and square) is indicated in the schema at the bottom. * = significantly different (*P*<0.05, by Wilcoxon rank sum) responses to either object (between summed power for the six sectors when the cross is in front versus the opposing sectors when the square is in front of the fly; See Methods). Σ Power, summed power of all frequencies in said frequency domain. N = 4 flies per genotype. Baseline responses for the 10 s immediately preceding novelty are shown in Figure S3.

There are limits to how much behavioral assays can reveal about attention-like processes in animals. In contrast, electrophysiology can provide detailed insight with regard to the temporal dynamics of selection and suppression of brain responses to competing stimuli. We presented the mutants *D0264*, *D0067*, and *D0177* with competing visual choices (a cross versus a square) while we recorded local field potentials (LFPs) from their brains using a previously reported paradigm to study visual selective attention in flies (Figure 3A, and see Methods). In previous studies, LFP responses to visual stimuli using this paradigm revealed that certain frequency domains within the LFP, centered on the 20–30 Hz range, were correlated with visual salience [2]. In this paradigm, wild-type brains display increased LFP activity for novel objects while, at the same time, exhibited suppressed LFP activity for competing non-novel objects, following 100 s of visual training [3]. *dunce* mutants fail to show any such LFP selection/suppression in the 20–30 Hz range, but, instead, show a smaller effect only at a lower (∼10–20 Hz) frequency range [3]. We first investigated baseline LFP responses to individual objects (a cross or a square) in our selected LTM mutants. The three strongest optomotor performers (*D0264*, *D0067*, and *D0177*) all revealed attenuated brain responses when exposed to the visual objects alone compared to wild-type flies (Figure 3B). To compare these responses to another LTM mutant, we conducted the same experiments on *A0023*, which had wild-type optomotor responsiveness and yet was not distractible (Figure 2D). Surprisingly, *A0023* also showed attenuated brain responsiveness compared to wild type (Figure 3B). Thus, the four LTM mutants tested all appear to respond less strongly to visual objects (like *dunce^1^* [3]) regardless of their differences in visual behavior.

LFP responsiveness to novelty was investigated by presenting distinct objects together following a training period during which flies were exposed to identical objects (Figure 3C). The three mutants *D0264*, *D0067*, and *D0177* revealed different responses to novel objects at the level of brain LFP activity, and none exhibited wild-type responses (Figure 3D, and see Figure S3 for baseline responses prior to novelty). *D0264* responded strongly in the 10–20 Hz range, with some responsiveness in the 20–30 Hz range. *D0067* responded weakly in the 10–20 Hz range only, resembling *dunce^1^* LFP effects. *D0177* responded best in the 30–40 Hz range. The weakest effect was thus found in the mutant that proved least distractible in our behavioral assay, *D0067*. To test if the distraction phenotype (Figure 2) might predict responsiveness to novelty in our brain recording preparation, we tested an additional LTM mutant, *A0023*, which was also found to be unaffected by a distracter in the behavioral paradigm. Indeed, LFP responsiveness to novelty in *A0023* was also defective, most resembling *D0067* by the absence of a strong frequency component in the LFP response (Figure 3D). Thus, LTM mutations appear to influence the frequency of LFP oscillations in the fly brain as well as visual behavior.

The selective response to novelty depends on the amount of prior exposure to the non-novel object [3]. Wild-type flies require at least 50 s of visual training (or exposure) to identical squares to produce a selective response to a novel cross and a suppression of a response to the competing non-novel square. 25 s fails to generate such a response in wild type. To confirm the LFP frequency effects found in *D0264*, *D0067*, and *D0177*, we repeated the novelty experiments with 50 or 25 s of training. None responded significantly after 25 s training, while 50 s of training again yielded significant responses in the 10–20 Hz range for *D0264* and in the 30–40 Hz range for *D0177* (Figure S4). The effect on LFP frequency ranges in these strains therefore appears to be robust, whereas, in *D0067* (like *dunce*), the effect is weak.

### Spatiotemporal rescue of *dunce*

**Figure 4:**
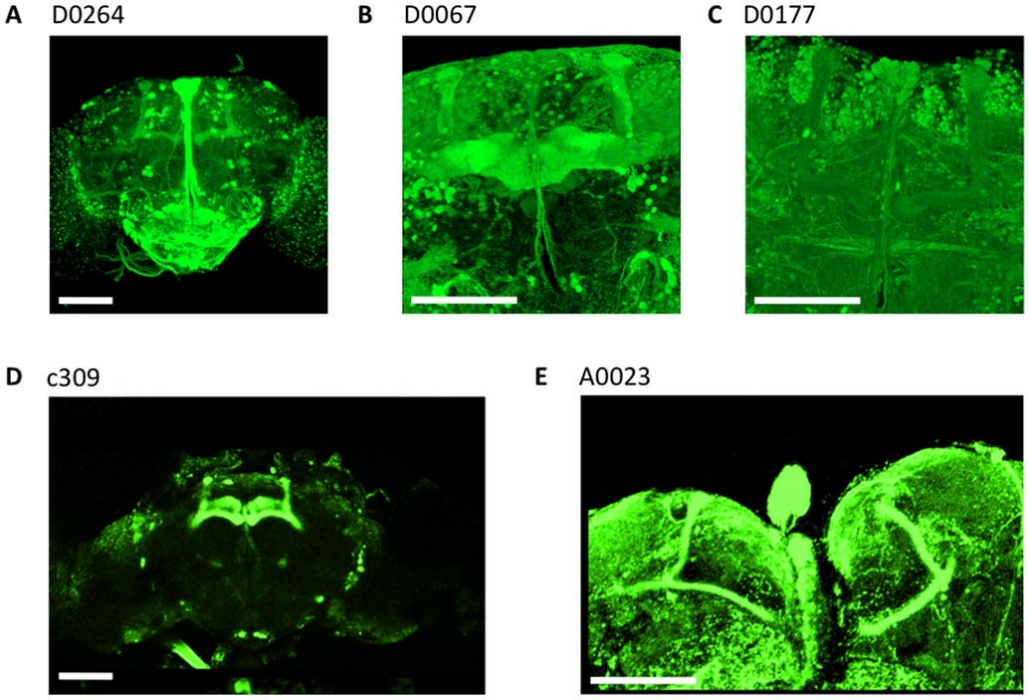
GFP expression in the Gal4 circuits studied. A. *D0264-*Gal4*/*UAS*-GFP* B. *D0067-*Gal4*/*UAS*-GFP* C. *D0177-*Gal4*/*UAS*-GFP* D. *c309/*UAS*-GFP* E. *A0023-*Gal4*/*UAS*-GFP*. Images A–C provided by Ann-Shyn Chiang. The scale bar is 100 µm.

We made use of the Gal4/UAS system [13] to explore the neuroanatomy of brain circuits which could be subserving the attention-like phenotypes described above. *D0264*, *D0067*, and *D0177* are homozygous Gal4 inserts on standardized genetic backgrounds (see Methods), and as such can be used to explore the function of circuits impacted by the enhancer trap in addition to the mutagenic effects caused by the transposable element. We have previously shown that *dunce^1^* attention defects can be rescued by expressing wild-type *dunce* in the brain throughout development [3]. *dunce*-like optomotor phenotypes in *D0264*, *D0067*, and *D0177* suggest that these mutations might compromise brain circuits relevant to *dunce* function (*i.e.*, they may represent downstream plasticity genes expressed in the same cells as *dunce*). We thus sought to examine the brain regions required to rescue *dunce^1^* defects by expressing wild-type *dunce* in the *D0264*, *D0067*, and *D0177* cells, in a mutant *dunce^1^* background. It is important to emphasize that in this heterozygous context, the LTM mutants become simple Gal4 drivers; the three inserts are recessive and have no abnormal phenotype as heterozygotes over wild type (data not shown). In the current UAS/Gal4 scenario, we are therefore testing effects on the brain cells controlled by these insertions, not the effects of the insertions themselves. Whether these Gal4-expressing cells may be involved in olfactory learning and memory has not been formally investigated. However, the presence of mushroom body labeling for many of these Gal4 insertions [8] (and see Figure 4) suggests olfactory memory circuits may be involved.

**Figure 5:**
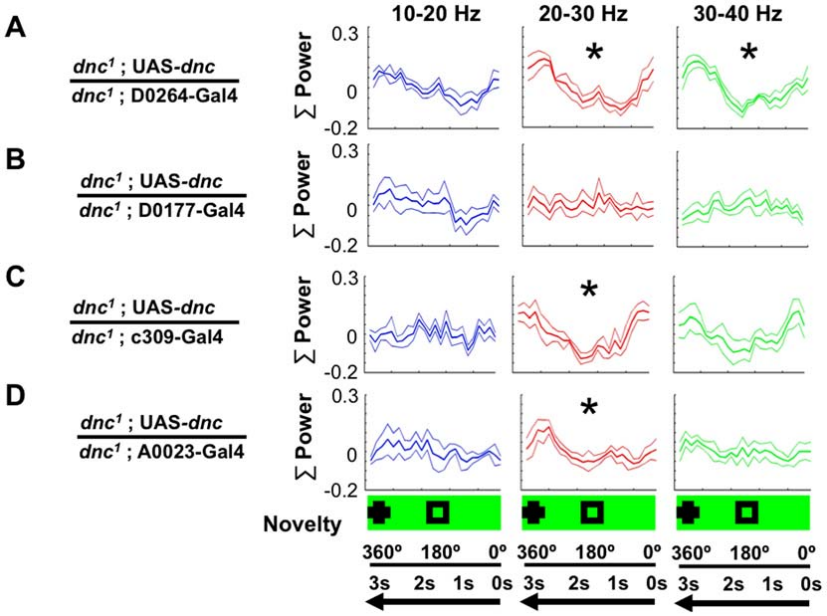
*dunce^1^* spatial rescue. A. Wild type *dunce* expression in the *D0264-Gal4* cells, in a mutant *dunce* (*dnc^1^*) background, produced significant (*, *P*<0.05, by Wilcoxon rank sum) responses to novelty across two frequency domains. The same novelty paradigm as in Figure 3 was used. N = 5 flies. B. *dunce* expression in the *D0177-Gal4* cells did not rescue novelty responses in any of the 3 frequency domains. N = 5 flies. C. *dunce* expression in the *c309* circuit rescued the novelty responses in the 20–30 Hz frequency domain. N = 4 flies. C. *dunce* expression in the *A0023-Gal4* cells rescued the novelty responses in the 20–30 Hz frequency domain. N = 4 flies. Baseline responses immediately preceding novelty are shown in Figure S5.

Expressing wild-type *dunce* in the Gal4 cells defined by *D0264* (Figure 4A) rescued *dunce^1^* LFP defects: the *dunce^1^; UAS-dunce/D0264-Gal4* strain responded strongly to novelty with a characteristic 20–30 Hz selection/suppression profile following 100 s of visual training (Figure 5A, and see Figure S5 for baseline responses). In contrast, the *dunce^1^; UAS-dunce/D0177-Gal4* strain failed to rescue *dunce^1^* defects in this paradigm: we saw a small and insignificant response only in the 10–20 Hz range, like *dunce* mutants (Figure 5B). Rescue experiments for *D0067*-*Gal4* were not possible as the rescue strain *dunce^1^; D0067-Gal4/UAS-dunce* was larval-lethal, possibly due to widespread expression in the brain beyond the mushroom bodies (Figure 4B). The *D0177* driver appears to not label in the Kenyon cell processes of the mushroom bodies (Figure 4C), so it is perhaps not surprising that this enhancer trap fails to rescue *dunce^1^*. The enhancer traps *D0067* and *D0264* express in neurons throughout the *Drosophila* brain, with some overlap in the α and β lobes of the mushroom bodies (Figure 4A, B). Most striking in *D0264* is strong expression in the pars interecerebralis, with projections via the median bundle to the suboesophageal ganglion. A similar pattern of expression (minus the mushroom bodies) has been found in the *c767-Gal4* driver for neurons controlling EGFR signaling and sleep in *Drosophila* [14].

To investigate more specifically whether the mushroom bodies might be involved in *dunce*-mediated visual attention responses, we used a Gal4 driver labeling most of the mushroom bodies, *c309-Gal4* [15] (Figure 4D). Expressing the wild-type *dunce* gene in the *dunce^1^; UAS-dunce/c309-Gal4* strain rescued LFP responsiveness to novelty (Figure 5C). The involvement of the mushroom body was further supported by significant novelty detection in the strain *dunce^1^; UAS-dunce/A0023-Gal4* strain (Figure 5D). In addition to widespread expression throughout the brain, *A0023-Gal4* appears to also label a more restricted subset of cells in the α and β lobes of the mushroom bodies compared to *D0264-Gal4* (Figure 4E). However, *dunce^1^* rescue with *A0023-Gal4* was only marginally significant (*P* = 0.05) compared to *c309-Gal4* and *D0264-Gal4* rescue. Consistent with this, optomotor behavior was rescued in *dunce^1^; UAS-dunce/c309-Gal4* (OI = 0.55±0.14) but not in *dunce^1^; UAS-dunce/A0023-Gal4* (OI = 0.95±0.25) or *dunce^1^; UAS-dunce/D0177-Gal4* (OI = 1.05±0.18). *dunce^1^; UAS-dunce/D0264-Gal4* was not testable for behavior in the maze as animals refused to enter.

**Figure 6:**
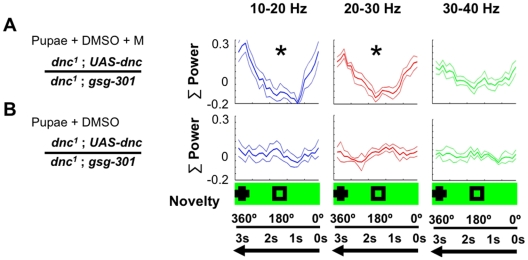
*dunce^1^* temporal rescue. A. Injection of Mifepristone (M) in pupae (see Methods) to activate *dunce* expression throughout mutant *dnc^1^* brains rescued (*, *P*<0.05, by Wilcoxon rank sum) the selective novelty response across two frequency domains. N = 5 flies. B. Injection of only the vehicle (DMSO, see Methods) in pupae of the same strain as in A did not produce any significant novelty responses in adult flies. N = 5 flies. Baseline responses immediately preceding novelty are shown in Figure S6.

Having spatially narrowed *dunce* effects on attention-like phenotypes to potentially the α and β lobes of the mushroom bodies, we next investigated the temporal window required for *dunce* function in our selective attention paradigm. We have previously shown that *dunce* was required throughout development in order to rescue attention-like defects in the adult by inducing wild-type *dunce* in a mutant background in larvae (induction in the adult did not rescue the defects [3]). We now asked whether attention-like defects could be rescued by expressing wild-type *dunce* only in the late pupal stage of development. To test this, we injected *dunce^1^; UAS-dunce/gsg-301* pupae with Mifepristone to induce wild-type *dunce* expression (see Methods) and then tested adults for brain responses to visual novelty. We found that expressing *dunce* at a late pupal stage was sufficient to rescue attention-like phenotypes in the brain (Figure 6A, and see Figure S6 for baseline responses). In contrast, injection of only the vehicle (DMSO) into late-stage pupae did not rescue *dunce^1^* attention defects (Figure 6B). The *dunce* protein is thus required at earliest during pupal brain development for enabling subsequent attention-like responses in the adult. Taken together with our spatial rescue experiments, this result suggests that mushroom body Gal4 expression patterns in the pupal or early adult brain may be more relevant to understanding *dunce* rescue effects than patterns in the adult brain.

### Active control of attention/memory neurons

**Figure 7:**
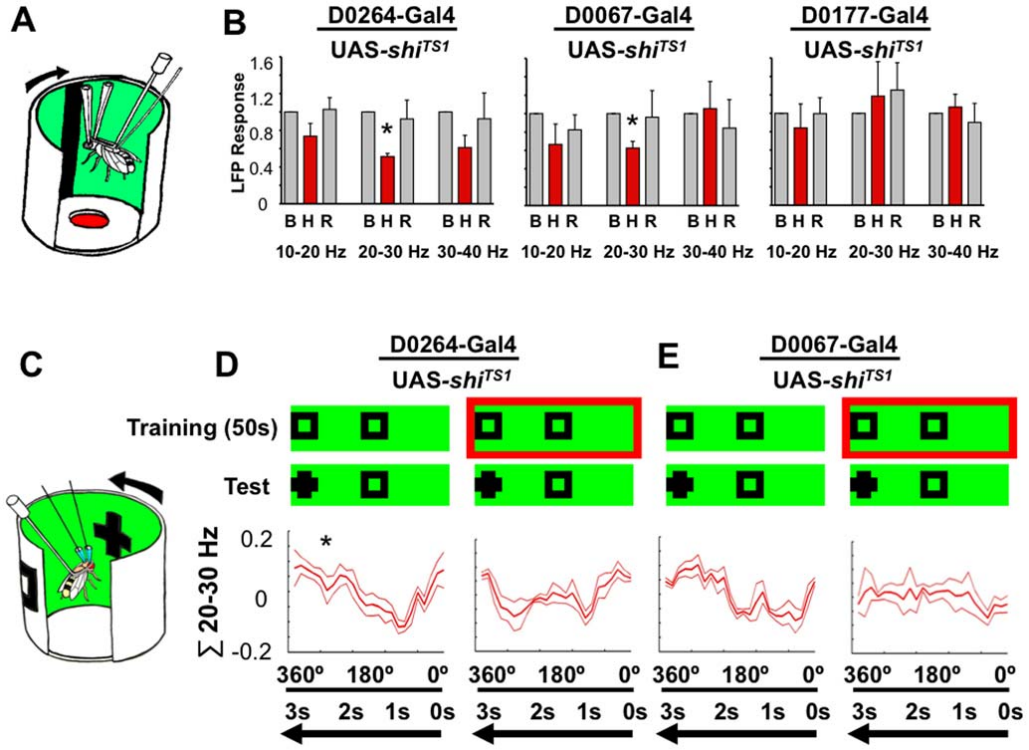
Electrophysiology of synaptic silencing in three Gal4 circuits. A. Recording arena setup. The visual stimulus is a moving dark bar on a lit green background. B. Brain responses to the visual for the *D0264*, *D0067*, and *D0177* complemented by *UAS-shibire^TS^*. LFP responses are calculated as the normalized maximum – minimum for three successive temperature conditions [2], for three frequency domains as indicated. B = baseline response, at 22°C, H = response during heating at 38°C, R = recovery response at 22°C. Calculations were for 100 s at each condition, averaged from a triplicate test per fly. N = 4 flies per genotype. * = significantly different from baseline, set at 1 (*P*<0.05, by *t*-test). C. Recording arena setup. D. The same paradigm as in Figure 3, applied to *D0264-*Gal4/UAS-*shibire^TS^*. Left panel: control response to novelty after 50 s of training. *, significant response (±s.e.m.) to the novel cross (*P*<0.05, by Wilcoxon rank sum). Right panel: Flies were heated at 38° during training (red box) and returned to 22°C during testing for novelty responses. The test was repeated 5 times for averaging. N = 5 flies E. The same experiment for the strain *D0067-*Gal4/UAS-*shibire^TS^*, N = 4 flies.

If select mushroom body circuits rescue *dunce^1^* attention defects, then silencing synaptic output from these structures should modulate brain activity correlates of attention. Neuroanatomical localization of attention-like behavior was further explored by transiently silencing synaptic activity via the *D0264*, *D0067*, and *D0177* Gal4 drivers, complemented by experiments more restricted to the mushroom bodies using other Gal4 drivers. LFP oscillations such as the 20–30 Hz response to visual salience are most likely generated by synchronization of synaptic potentials. This implies that “upstream” neurons are synchronizing their output in order to generate summed electrical effects near the recording site. Thus, modulating LFP activity by silencing distinct neuronal circuits should narrow the sources of neuronal synchronization resulting in the 20–30 Hz response to novelty. To attenuate synaptic output we expressed the *shibire^TS^* gene in the three Gal4 networks defined by *D0264*, *D0067*, and *D0117*, as well as in *A0023*, *c305a*, and *c739*, which appear to label subdivisions of the mushroom bodies, along with diverse other structures [16] (and see Figure S7). *UAS-shibire^TS^* expresses a thermolabile form of dynamin, involved in vesicle recycling at synapses, and causing rapid neurotransmitter depletion at the restrictive temperature (>30°C) [17]. To test the *shibire^TS^* effects in defined Gal4 expressing cells, we simplified the visual paradigms to a single moving bar (Figure 7A), which evokes a baseline LFP response that is attenuated by *c309* expression of *shibire^TS^* [2]. When *shibire^TS^* was expressed in the *D0264* Gal4 cells (Figure 4A), LFP activity was attenuated at the restrictive temperature for all frequency ranges between 10 and 40 Hz. However, only the effect at 20–30 Hz was significant (Figure 7B, left panel). After this transient attenuation, LFP responses returned to baseline levels upon recovery. Interestingly, attenuating synaptic release in the *D0264* cells also immobilized the flies (data not shown), presumably due to widespread expression with this driver (see Figure 4A), so we were not able to test these animals behaviorally. *shibire^TS^* effects on the *D0067* cells were similar to *D0264* with regard to brain responses, again revealing specificity for the 20–30 Hz frequency range which was attenuated at the restrictive temperature (Figure 7B, middle panel). In contrast, expressing *shibire^TS^* in the *D0177* cells did not compromise LFP activity when synaptic release was attenuated in those cells (Figure 7B, right panel). The relevance of the mushroom bodies for this response was further investigated with three strains showing more restricted expression in these structures than *c309*. Restricting *shibire^TS^* effects to the *A0023-Gal4* cells failed to attenuate the LFP response, but restricting the same effects to the *c305a* and *c739* cells attenuated the brain response (Figure S7). Finally, mushroom body effects on brain activity were matched by behavioral data: *c309*/*UAS-shibire^TS^*, *c305a*/*UAS-shibire^TS^*, and *c739*/*UAS-shibire^TS^* all displayed significantly increased optomotor responsiveness following exposure to the restrictive temperature (see Methods) (*c309*/*UAS-shibire^TS^*: OI = 1.86±0.17 heated versus 0.77±0.07 baseline; *c305a*/*UAS-shibire^TS^*: OI = 1.23±0.14 heated versus 0.67±0.15 baseline; *c739*/*UAS-shibire^TS^*: OI = 1.16±0.23 heated versus 0.61±0.07 baseline, N = 4 maze runs for each). These behavioral results are consistent with our view that the mushroom bodies are involved in visual suppression; optomotor responsiveness increases (and 20–30 Hz brain responsiveness decreases) when mushroom body output is compromised.

The 20–30 Hz response has been associated with visual attention [2], [3]. For flies to respond appropriately to novelty (as shown in Figures 3, 5 & 6) presumably requires a level of attention (as opposed to mere exposure) to the visual objects being presented during training (*e.g.*, the two squares). Abolishing attention mechanisms during training should thus abolish the selective novelty response. This was indeed the case for two of the Gal4s described above that specifically abolish the 20–30 Hz response (*D0264* and *D0067*). We exposed *UAS-shibire^TS^*/*D0264-Gal4* and *UAS-shibire^TS^*/*D0067-Gal4* animals to the restrictive temperature only during training in our visual novelty paradigm (Figure 7C). For both lines, attenuating synaptic output during training resulted in a loss of the selective 20–30 Hz response to visual novelty, with flies responding to either competing object equally, as for naïve flies (Figure 7D,E, right panels). Control experiments in the same animals done without heating showed the characteristic 20–30 Hz selection/suppression profile following novelty (Figure 7D,E, left panels). This suggests a possible role in memory acquisition for the circuits embedded within these Gal4 expression patterns and further emphasizes that attention and memory are likely to involve interacting processes in the fly brain.

## Discussion

To remember something well, it helps to pay attention. Studies of selective attention have traditionally been restricted to higher animals such as humans and monkeys, and as a result, our understanding of the phenomenon has often been confounded by our lack of understanding of the nature of consciousness and its relation to attention. Yet, attention may be better studied in other animals as essentially a suppression phenomenon, where the brain filters out most of the world except those stimuli immediately relevant or salient to the behavioral task at hand. In this view, in order to study attention we must measure suppression effects. This concept underpins the behavioral approach we used to uncover mutants with attention-like defects: specifically, we suggest that mutants such as *dunce^1^* display increased optomotor responsiveness because they are less able to suppress visual reflexes, and that this is in direct relation to their attention-like defects.

How an animal's capacity for visual suppression becomes a dynamic, experience-dependent phenomenon akin to selective attention seems to be a more difficult problem, requiring a parallel understanding of memory circuits and their interaction with suppression mechanisms. Understanding this interaction is possible through the use of model organisms, such as *Drosophila melanogaster*, where we can manipulate memory circuits to examine their effects on suppression. In this study, by progressing from behavioral screens to electrophysiology and active control of selected groups of neurons, we showed that 1) A sub-group of olfactory learning and memory mutants in *Drosophila* are also characterized by more general defects in visual attention-like processes, and 2) the mushroom bodies, structures traditionally associated with olfactory learning and memory, seem to also be involved in modulating visual attention.

Two LTM mutants clearly stood out by displaying optomotor response levels equivalent to *dunce* mutants: *D0264* and *D0067*. Our strategy of screening for high optomotor levels was successful as both high optomotor performers revealed defects at the level of brain activity: LFP responsiveness was either severely compromised (in *D0067*) or LFP power was shifted to a lower frequency domain (in *D0264*). Since these mutants are also enhancer trap Gal4 strains [8], we were able to show that the cells subserved by these selected elements also attenuated the 20–30 Hz LFP response to visual salience when tested in combination with *UAS-shibire^TS^* at the restrictive temperature. The specificity of the effect for the 20–30 Hz range was striking (10–20 Hz and 30–40 Hz were only moderately affected), suggesting that this narrow frequency band at 20–30 Hz is functionally associated with activity in these networks of neurons. However, *D0264* and *D0067* seem to affect rather distinct groups of cells, with only the mushroom body (MB) α and β lobes representing a clear overlap between the two.

In a previous study, we found other MB-expressing Gal4 strains that, when driving UAS-*shibire^TS^*, attenuated the 20–30 Hz response, namely *c309* and *121y* [2]. To narrow down which parts of the MBs might be required for generating the LFP, we conducted additional UAS-*shibire^TS^* experiments in the present study on two more Gal4 strains shown to target the α and β and α' and β' lobes of the MB, *c739* and *c305a*, respectively [16]. Both attenuated the 20–30 Hz response, but not as specifically as *D0264* and *D0067*; neighboring frequency domains were also attenuated (Figure S7). In contrast, the absence of any LFP attenuation with *A0023* (which expresses only in a small subset of the MB Kenyon cells as well as much of the rest of the brain) suggested that a minimal portion of the MB is required for the LFP response. Thus, a directed approach to MB anatomy as well as an indirect approach based on olfactory LTM mutants all appear to point to the MB playing a key role in generating this visual salience-related LFP. The more focused effect of the *D0264* and *D0067* drivers to a narrow LFP frequency domain suggests that specificity in the LFP response is tied more to widespread circuits including much of the MB, possibly interacting with other brain regions, rather than to the sole contribution of individual MB lobes. Finally, our data showing that optomotor responsiveness increases when synaptic output is transiently blocked in *c309*, *c305a*, and *c739* cells is consistent with our view that the MBs are involved in visual suppression mechanisms.

In this study we have thus narrowed the search space for control of visual attention-like phenotypes in the fly. First, it is clear that a proportion of learning and memory mutants found by completely different (olfactory) paradigms have relevant defects here. Therefore, the significant resources required for accomplishing a blind forward genetic screen may be sidestepped by exploring existing memory mutants. Again, a common feature of these mutations is the relevance of the MB to cognitive function. All the memory mutants we have found that compromise attention-like phenotypes target MB function: *radish* (van Swinderen and Brembs, submitted), *dunce*, *rutabaga*, *D0067*, and *D0264*. Consistent with this trend, synaptic silencing of the MB attenuates attention-like responses in the brain, which suggests that the MB neurons are “upstream” of the circuits synchronizing to produce the LFPs detected in the brain. Since some Gal4 drivers were able to both rescue *dunce^1^* as well as attenuate 20–30 Hz responses, this suggests that *dunce* function overlaps to some extent with neurons causing the LFP oscillations (e.g., in *D0264*), but not always (e.g., in *A0023*). Immunohistochemical studies have shown that *dunce* is primarily expressed in the MB [19], which is consistent with our results showing rescue in these structures. Furthermore, Gal4 drivers such as *c309* (which rescued *dunce^1^* function) are already active during metamorphosis [20], with strong expression in the MB of pupae (data not shown). Thus, we propose that *dunce* function during pupal development is required to generate the MB wiring necessary for attention-like oscillations. Whether the MB neurons themselves are oscillating (as opposed to causing oscillations downstream) is an open question. For the MBs to be required for generating oscillations while also generating the LFP responses themselves (as suggested by our *dunce^1^* rescue experiments) suggests a feedback system within these structures, as has been posited for other aspects of MB function [21]. Finally, our rescue of *dunce^1^* phenotypes by expressing wild-type protein in the pupal stage is consistent with a role for the MB here: substantial MB neuronal proliferation and organization occurs during this stage [22]. It is unclear why rescue of olfactory memory phenotypes can occur after this phase in development (albeit, for *rutabaga* mutants [23]) whereas visual attention phenotypes require pupal development (this study, and [3]). Future experiments investigating both visual attention and olfactory memory while controlling gene expression from pupae to adulthood (e.g., gene knockdowns or combined Gal4/Gal80 experiments [9] allowing for Gal4 induction only in the adult) should begin to unravel the level of overlap between visual attention and olfactory memory phenotypes with regard to mushroom body development and function.

Why might the MBs be relevant for selective attention in the fly? The consensus over several years of study is that these structures are crucial for olfactory learning and memory in flies [21]. Visual learning appears to reside in another brain area, the central complex [24]. However, while visual studies point to the MB as being dispensable for simple aversive visual learning [25] more recent studies have shown the MB to be required for contextual learning [26], [27], [28], [29]. This “more difficult” form of learning includes context generalization, involving binding between modalities, disambiguating contradictory cues, and extracting salience from background. All of these phenomena are hallmarks of attention in human studies; context generalization, for example, may be re-interpreted as the suppression of non-predictive competing stimuli. The fact that the MB are required for these aspects of visual learning is consistent with their potential role in selective attention. Thus, we propose that these structures house a gating mechanism to memory formation, which could then be distributed throughout the brain (including the MBs themselves). The gating mechanism would involve a dynamic selection of behaviorally relevant stimuli and suppression of competing stimuli, as we have been measuring in this study. Defective suppression mechanisms would thus lead to the dominance of certain prepotent behaviors (e.g., the optomotor response) and possibly learning defects when these prevent proper acquisition (or retrieval) of relevant events. Although we did not test visual learning in the Gal4 networks described in this work, a recent publication using aversive phototaxic suppression (APS) found that wild-type *dunce* expression in *c309* cells rescued *dunce* learning defects in that visual paradigm [7]. The inability of *dunce* mutants to suppress a prepotent response, and rescue by wild-type *dunce* expression in the MB is entirely consistent with results from our study.

We propose that brain oscillations generated in part by the MB provide a mechanism of stimulus selection or suppression. Populations of neurons potentially firing in response to a stimulus (e.g., a moving grating) are either enabled or suppressed, depending on spatio-temporal characteristics of the 20–30 Hz oscillation. The connection between these attention-like oscillations and memory formation would be through spike timing dependent plasticity (STDP) in MB circuits, as has been demonstrated in another insect system [30]. In that study, oscillations in the locust MB were found to play a key role in synaptic plasticity mechanisms. This matches our observation that the three LTM mutants uncovered by our optomotor screen all display aberrant oscillatory behavior in the brain. Active control of such oscillations in *Drosophila* should further unravel the connection between attention and memory systems in the insect brain.

## Materials and Methods

### *D. melanogaster* strains and stocks

Flies were cultured at 22°, 50%–60% humidity, 12 hr:12 hr light:dark cycle on standard media. Wild-type flies are from the Canton-S strain; the *dunce^1^* mutant was obtained from Leslie Griffith (Brandeis University), the *gsg-301* strain from Scott Waddell (University of Massachusetts), and the 36 LTM mutants from Josh Dubnau (Cold Spring Harbor Labs). The strains *D0264*, *D0067*, and *D0177* were outcrossed to a *white^+^* background by standard procedures. Double mutants between these LTM strains and *dunce^1^* mutants were made by standard procedures using chromosomal balancers. Only 2–7 day-old female flies were tested, one day after having been anesthetized with CO_2_.

### Optomotor maze and population responses

Visual responsiveness was tested behaviorally using an 8-choice maze placed over a CRT computer screen. The maze paradigm used was exactly as described previously [1]. Flies were collected under CO_2_ anesthesia the day before an experiment and loaded (N = 25–30) into disposable polyethylene “jumbo” transfer pipettes (Fisher Scientific), where they were allowed to acclimatize 3 min in the dark before the pipette was inserted into to maze's starting position. After running the choice maze (2–5 min) in a darkened room, the flies' distribution among the nine collection tubes was scored as a weighted average ranging from −4 to +4 (see Figure 1). The Optomotor Index (OI) is the deviation of the weighted average from 0, the middle tube, where positive scores indicate optomotor responses in the same direction of image motion. All statistics were *t*-tests of experimental means (following tests for normality), unless otherwise specified (Wilcoxon rank sum tests were used for non-normally distributed data).

Visual stimuli presented to flies running the maze were exactly as described previously: 1 cm green/black gratings moving at 3 Hz, or single competing static black bars pasted onto the inside of the box covering the maze [1]. Visual distraction tests were conducted to assess how competing static images (such as the black bars) could compete with the moving green/black grating for perceptual resources. Tests for visual distraction required four separate experiments, each performed 8 times on separate fly groups within a genotype (see Figure 2B): Responsiveness to the black bar alone over a static green/black grating. G: Responsiveness to the moving grating alone without the black bar. GB+: Responsiveness to the moving grating when the bar is on the side towards where the grating is moving. GB-: Responsiveness to the moving grating when the bar is on the opposite side of grating movement. Significant distraction was determined by performing *t*-tests between the GB+ condition and the GB- condition, with the threshold level for significant differences where *P*<0.05. For experiments testing Gal4/UAS-*shibire^TS^* effects on optomotor responsiveness, we pre-incubated flies for 10 minutes at 37°C (already in their loading tubes) and then immediately ran them in the maze. Resulting optomotor scores were compared (by *t-*test set at *P*<0.05) to baseline (22°C) runs of the same genotypes. Wild-type optomotor responsiveness was unaffected by pre-incubation at 37°C (OI = 0.57±0.17, compared to OI = 0.78±0.09).

### Visual learning paradigms

We employed two different paradigms to assay visual learning in walking flies. In the first paradigm, we used the optomotor maze to test for changes in performance when groups of flies re-ran the maze multiple times in immediate succession. Each individual fly was scored (−4 to +4) as it completed the maze and then immediately collected into a loading tube. Upon collecting the requisite number of flies for an experiment (25–30), these were then immediately re-tested in the same maze for up to four successive runs. We found that a form of learning (measured by increased optomotor responsiveness) reached a plateau after two re-runs in wild-type flies. Analyses of optomotor responsiveness were performed exactly as for regular maze experiments.

In the second visual learning paradigm, we tested associative conditioning by modifying a previously described protocol [11] where visuals are associated with aversive shaking. Flies (25–30) were loaded into glass test tubes attached to a vibrating device (www.neutrogenawave.com). The tubes formed a “V” connected at the center by the vibrator, such that flies could travel into either tube but were shaken down to the middle upon vibration (see Figure S1). The “V” was centered over a computer monitor where visuals could be displayed on either half. Training involved five 1 min sessions of shaking associated with one color which then alternated with five 1 min sessions without shaking associated with another color. Shaking epochs consisted of six 5 s periods without vibration followed by six 5 s vibration periods. Upon the completion of training, flies remained in the dark for 1 min and then were shaken for 5 s (whereupon they tumbled to the center of the “V”). They were then presented with both visual stimuli, one on either side. The experiment was filmed throughout under infrared illumination, and the proportion of flies choosing either arm of the “V” were counted at regular intervals: 1) pre-exposure to the visuals for 2 min, 2) after each 5 s epoch without shaking during training (and equivalent epochs for the alternate visual), and 3) for successive 10 s epochs upon completion when flies were presented with a choice of both visuals simultaneously. The colors used were violet versus cyan; all strains were tested for normal responsiveness to these colors in the maze (data not shown). All experiments were equally balanced and spatially alternated, with shaking associated with one color first, followed by association of the other color with the shaking (for a new set of flies). Four groups of 25–30 flies were tested per experiment (four glass “V's” attached to the vibrator), and a learning index was calculated as: # flies in unshaken side - # flies in shaken side)/total # flies. Control of visuals and shaking was accomplished automatically using LABVIEW software. A movie of the paradigm is available in Supporting Information (Movie S1).

### Electrophysiology

Brain recordings were performed as described previously [2], [3], [31]. Briefly, two glass electrodes were implanted into the fly brain, one in the left optic lobe and the other in the central brain. A voltage differential between these, filtered between 1 and 100 Hz, was sampled at 300 Hz using Labview software (National Instruments). Visual novelty experiments and the analysis of local field potential (LFP) data were performed as described previously [3]. Two distinct visual objects were used for novelty experiments, a cross and a square. These were displayed inside a circular arena as dark objects moving on a green LED background (see Figure 3). The objects rotated around the fly once every 3 s. Training involved exposure for a set time (e.g., 100 s) to two identical squares, after which one of the squares changed to a cross (the novel object), which we refer to as a “novelty transition”. Brain responsiveness in the epoch after a novelty transition was analyzed for selection or suppression of the competing distinct images. Briefly, a Fourier analysis was performed in Matlab (Mathworks) for a moving window resulting in power spectra assigned to 24 sequential sectors of a full panorama rotation. Significant responses to one or the other object was then determined by comparing summed power within a frequency domain between the opposing 6 sectors for either object (when it is in front of the fly). Significance was set at *P*<0.05 and tested by the Wilcoxon rank sum method for all comparisons, as sample sizes were small and often not normally distributed. Analysis of LFP data for synaptic silencing experiments involving *shibire^TS^* were performed as described previously [2]. The front-to-back difference in LFP activity (again, after calculating power across sequential 24 sectors) across the visual field determined the response to a single visual object, and this was quantified for heated epochs (100 s at 38°C) and contrasted to baseline (room temperature) for significance (*P*<0.05) by *t-*test and/or Wilcoxon rank sum.

### Pupal injections

Late-stage pupae were injected with Mifepristone (Sigma) in experiments aimed at rescuing *dunce^1^* attention defects during development. 13.8 nL of 50 mg/ml Mifepristone (Sigma) in DMSO was injected into pupae using a Nanoject II system (Drummond). Pupae were stuck to a piece of tape on a glass slide, which was then transferred to a regular food vial. In control experiments 13.8 nL of only DMSO was injected.

### Brain imaging

One week old female brains of Gal4/UAS-mCD8::GFP strains were removed by standard procedures in cold PBS and whole mounts were imaged immediately using a confocal fluorescence microscope. Images in Figure 5A–C were generously provided by Ann-Shyn Chiang (National Tsing Hua University, Taiwan); these were confirmed in our own laboratory strains.

## Supporting Information

Figure S1 Visual learning by classical conditioning. A. The conditioning apparatus (see Methods) seen from the front and from the side. B. Wild-type flies demonstrated learning by walking into a chamber illuminated by a color not associated with shaking following training (see Methods). Movement toward the non-shaken color is indicated by positive performance histograms. Performance index (PI) is (# flies in unshaken color - # flies in the shaken color)/total # flies. Pre-test (cyan or violet): 2 min of exposure to either color shown simultaneously (cyan and violet, displayed on a CRT monitor). Distributions (shown as PIs±s.e.m.) of visible flies at 10 s, 30 s, 60 s, 90 s, and 120 s are shown, with cyan preferences above and violet preferences below the abscissa. Training (grey): Distributions (shown as PIs±s.e.m.) of flies to either color shown alone, immediately after the shaking epoch. Memory test (black): Distributions (shown as PIs±s.e.m.) of flies between colors presented simultaneously, immediately after a shaking epoch in the dark, counted at 10 s, 30 s, 60 s, 90 s, and 120 s. * = significant learning (P<0.05, by t-test) compared to zero. N = 24 experiments, balanced with 12 for either color associated with shaking, 100 flies per experiment split evenly among 4 chambers. C. dunce1 performance, N = 16 experiments, balanced with 8 for either color associated with shaking, 100 flies per experiment. D. D0264 learning, N = 16 experiments, balanced with 8 for either color associated with shaking, 100 flies per experiment. E. D0067 learning, N = 16 experiments, balanced with 8 for either color associated with shaking, 100 flies per experiment. F. D0177 learning, N = 16 experiments, balanced with 8 for either color associated with shaking, 100 flies per experiment.
(6.00 MB TIF)

Figure S2 Visual learning in the maze. A. Flies completing a maze, exactly as in Figure 1, were immediately collected into a loading tube and re-run through the same maze in batches of 20–30 animals. Tube score, the proportion of flies in each of the nine collection tubes, with tube +4 being most in the direction of optomotor flow. First, second, and third runs are shown in blue, red, and green, respectively, for wild-type flies. B. Average Optomotor Index for each of the runs in A. *, P<0.05, by t-test against means. C. Maze re-runs for D0264. *, P<0.05, by t-test. D. Maze re-runs for D0067. E. Maze re-runs for D0177.
(3.00 MB TIF)

Figure S3 Baseline LFP responses with brain activity in the 10 s immediately preceding a novelty effect (as shown in Figure 3). In this case, LFP activity for 3 frequency domains are in response to two identical squares (shown in the schema at the bottom of each column). Comparisons of activity for the six sectors representing each object revealed no significant effects. D0177 showed a significant response (at 30–40 Hz), but it was not mapped to either object.
(3.00 MB TIF)

Figure S4 Training requirements for novelty response in the brain. A. D0264 was trained (exposed to two identical squares, as in Figure 3) for 100 s, 50 s, and 25 s. * = significant response (±s.e.m.) to the novel object in the 10 s following a novelty transition (P<0.05, by Wilcoxon rank sum of the 6 sectors for the square versus the 6 sectors for the box). Results are shown for the 10–20 Hz frequency range only, where D0264 displayed greatest responsiveness (Figure 3). B. D0067 responses, for the 10–20 Hz domain, following the three different training regimes. C. D0177 responses, for the 30–40 Hz domain (see Figure 3), following the three different training regimes.
(3.00 MB TIF)

Figure S5 Baseline LFP responses for spatial rescue of dunce1. Brain activity in the 10 s immediately preceding a novelty effect (as shown in Figure 5). In this case, LFP activity for 3 frequency domains is in response to two identical squares (shown in the schema at the bottom of each column). The data follows exactly the outline of Figure 5. A significant effect was found for the c309 circuit in the 20–30 Hz range (P<0.05, Wilcoxon rank sum), indicating that in this particular strain flies responded to one square more than the other, immediately preceding a choice between a square and a cross.
(3.00 MB TIF)

Figure S6 Baseline LFP responses for temporal rescue of dunce1. Brain activity in the 10 s immediately preceding a novelty effect (as shown in Figure 6). M = Mifepristone. No significant effects were detected.
(3.00 MB TIF)

Figure S7 Electrophysiology of synaptic silencing in 3 mushroom body circuits. A. Recording arena setup. The visual stimulus is a moving dark bar on a lit green background. B. Brain responses to the visual for the Gal4 drivers A0023, c305a, and c739 [16] complemented by UAS-shibire. LFP responses are calculated as the normalized maximum - minimum [2] for three successive temperature conditions, for three frequency domains. B = baseline response, at 22°, H = response during heating at 38°C, R = recovery response at 22°. Calculations were for 100 s at each condition, averaged from a triplicate test per fly. N = 3 flies per genotype. * = significantly different from baseline, set at 1 (P<0.05, by t-test). C. UAS-GFP expression of the Gal4 lines tested in B.
(3.00 MB TIF)

Movie S1 Movie file of segments from visual learning experiment. Visual classical conditioning apparatus.
(40.34 MB AVI)
