# Supplementary figures and images for: Shared Visual Attention and Memory Systems in the Drosophila Brain
Source: PLoS One. 2009 Jun 19;4(6):e5989. doi: 10.1371/journal.pone.0005989 (PMC2694981; doi:10.1371/journal.pone.0005989)

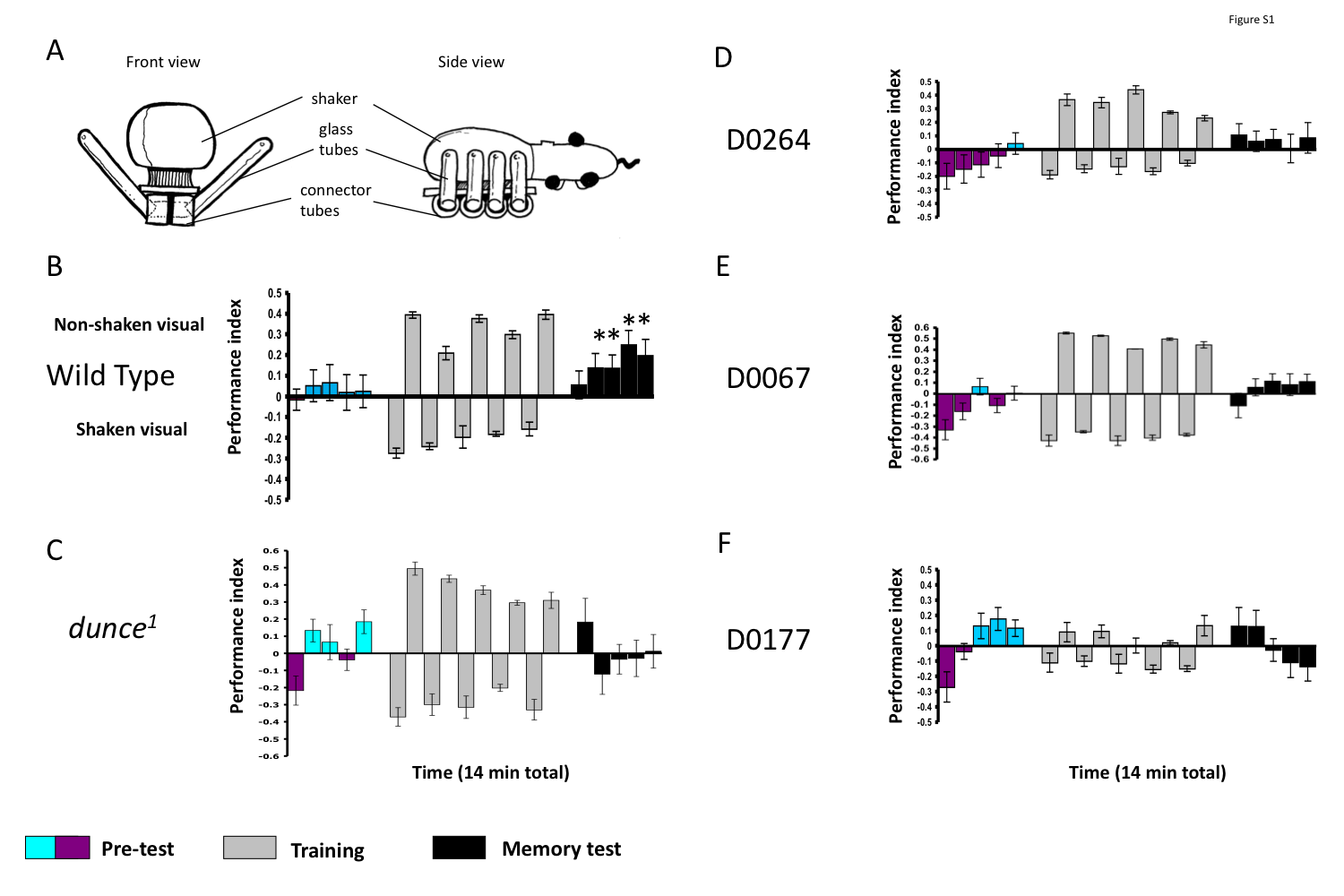

Supplement: Figure S1 — Visual learning by classical conditioning. A. The conditioning apparatus (see Methods) seen from the front and from the side. B. Wild-type flies demonstrated learning by walking into a chamber illuminated by a color not associated with shaking following training (see Methods). Movement toward the non-shaken color is indicated by positive performance histograms. Performance index (PI) is (# flies in unshaken color - # flies in the shaken color)/total # flies. Pre-test (cyan or violet): 2 min of exposure to either color shown simultaneously (cyan and violet, displayed on a CRT monitor). Distributions (shown as PIs±s.e.m.) of visible flies at 10 s, 30 s, 60 s, 90 s, and 120 s are shown, with cyan preferences above and violet preferences below the abscissa. Training (grey): Distributions (shown as PIs±s.e.m.) of flies to either color shown alone, immediately after the shaking epoch. Memory test (black): Distributions (shown as PIs±s.e.m.) of flies between colors presented simultaneously, immediately after a shaking epoch in the dark, counted at 10 s, 30 s, 60 s, 90 s, and 120 s. * = significant learning (P<0.05, by t-test) compared to zero. N = 24 experiments, balanced with 12 for either color associated with shaking, 100 flies per experiment split evenly among 4 chambers. C. dunce1 performance, N = 16 experiments, balanced with 8 for either color associated with shaking, 100 flies per experiment. D. D0264 learning, N = 16 experiments, balanced with 8 for either color associated with shaking, 100 flies per experiment. E. D0067 learning, N = 16 experiments, balanced with 8 for either color associated with shaking, 100 flies per experiment. F. D0177 learning, N = 16 experiments, balanced with 8 for either color associated with shaking, 100 flies per experiment. (6.00 MB TIF) [file pone.0005989.s001.tif]

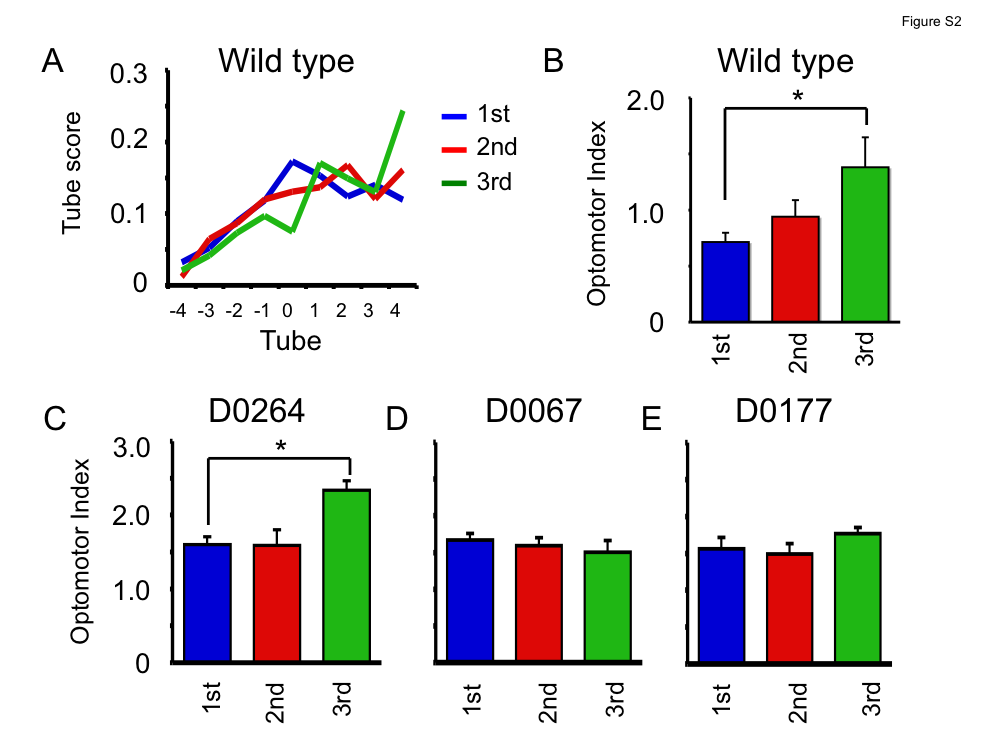

Supplement: Figure S2 — Visual learning in the maze. A. Flies completing a maze, exactly as in Figure 1, were immediately collected into a loading tube and re-run through the same maze in batches of 20–30 animals. Tube score, the proportion of flies in each of the nine collection tubes, with tube +4 being most in the direction of optomotor flow. First, second, and third runs are shown in blue, red, and green, respectively, for wild-type flies. B. Average Optomotor Index for each of the runs in A. *, P<0.05, by t-test against means. C. Maze re-runs for D0264. *, P<0.05, by t-test. D. Maze re-runs for D0067. E. Maze re-runs for D0177. (3.00 MB TIF) [file pone.0005989.s002.tif]

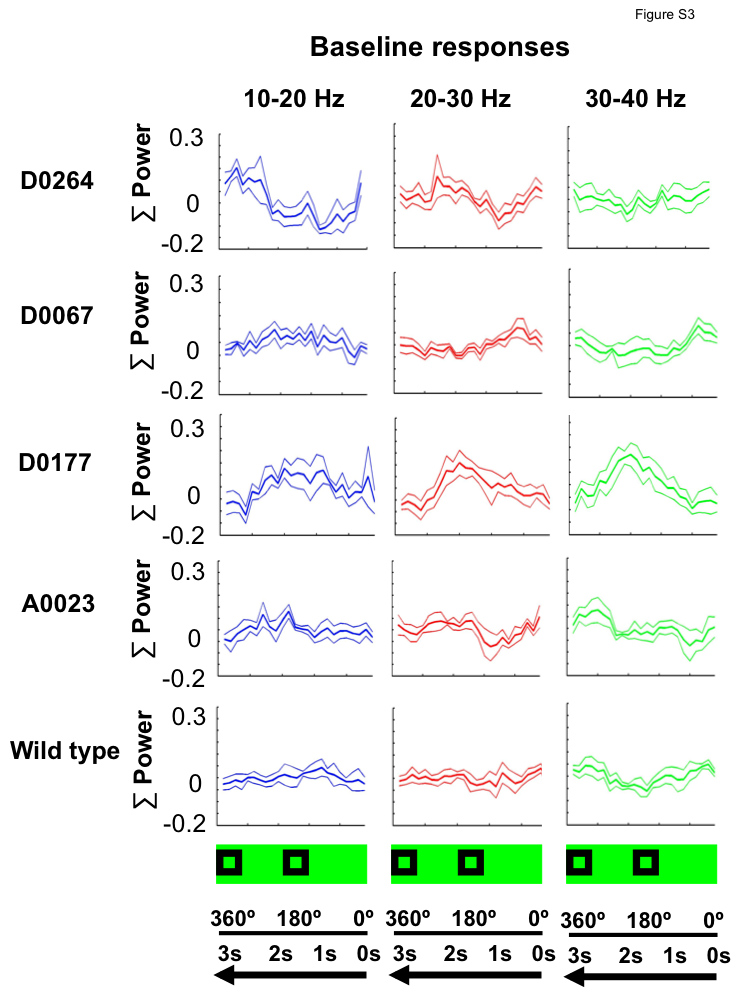

Supplement: Figure S3 — Baseline LFP responses with brain activity in the 10 s immediately preceding a novelty effect (as shown in Figure 3). In this case, LFP activity for 3 frequency domains are in response to two identical squares (shown in the schema at the bottom of each column). Comparisons of activity for the six sectors representing each object revealed no significant effects. D0177 showed a significant response (at 30–40 Hz), but it was not mapped to either object. (3.00 MB TIF) [file pone.0005989.s003.tif]

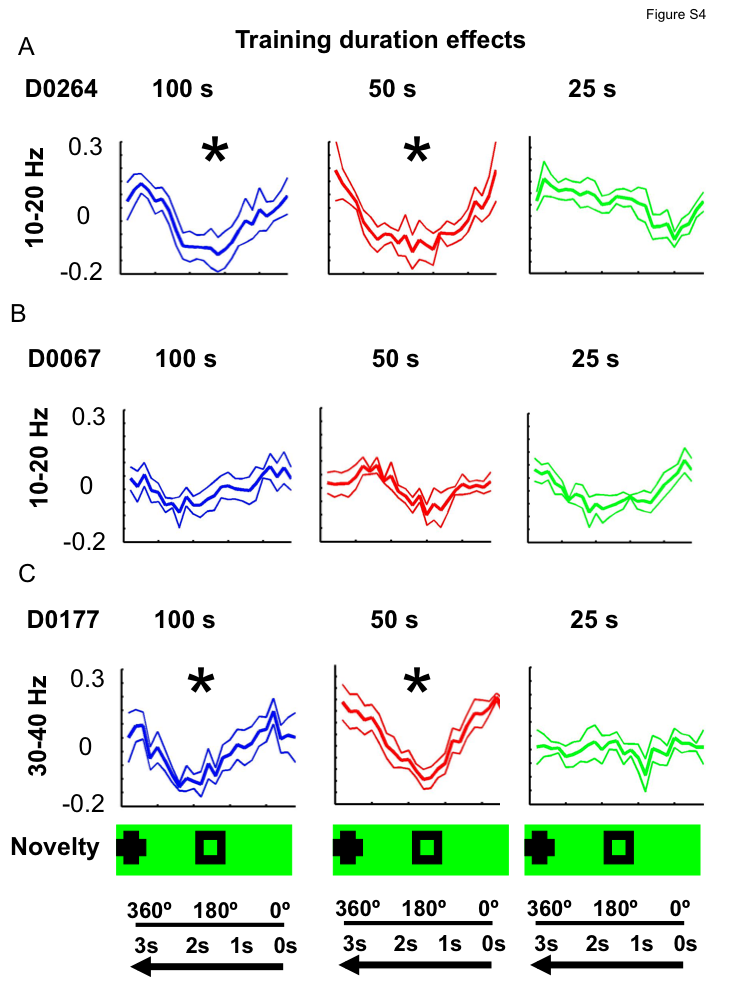

Supplement: Figure S4 — Training requirements for novelty response in the brain. A. D0264 was trained (exposed to two identical squares, as in Figure 3) for 100 s, 50 s, and 25 s. * = significant response (±s.e.m.) to the novel object in the 10 s following a novelty transition (P<0.05, by Wilcoxon rank sum of the 6 sectors for the square versus the 6 sectors for the box). Results are shown for the 10–20 Hz frequency range only, where D0264 displayed greatest responsiveness (Figure 3). B. D0067 responses, for the 10–20 Hz domain, following the three different training regimes. C. D0177 responses, for the 30–40 Hz domain (see Figure 3), following the three different training regimes. (3.00 MB TIF) [file pone.0005989.s004.tif]

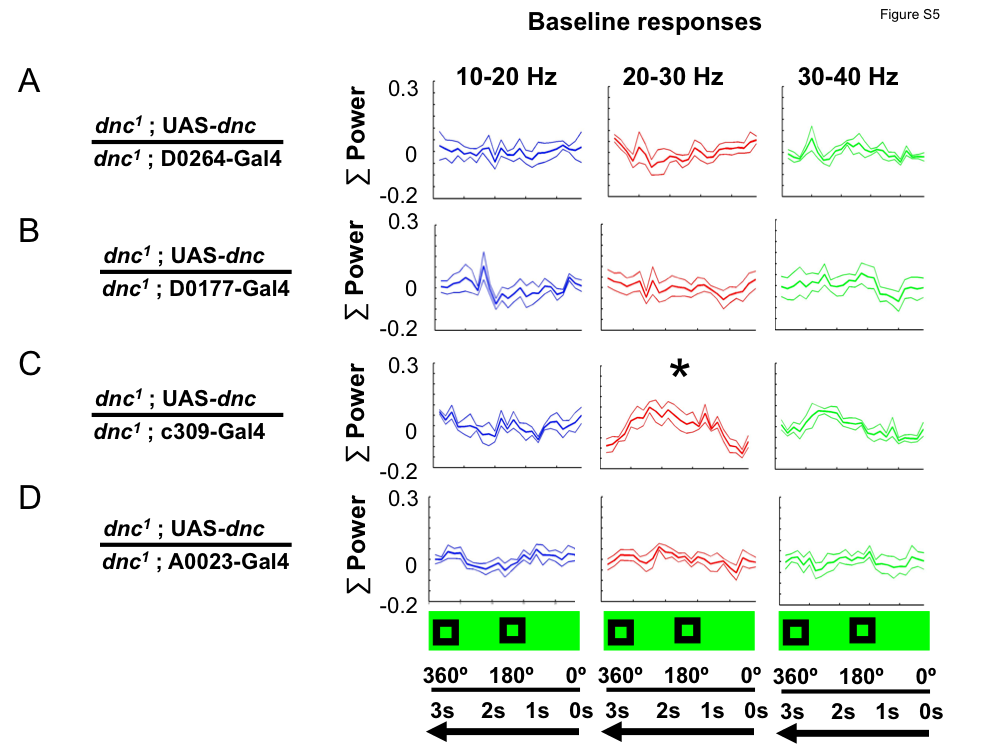

Supplement: Figure S5 — Baseline LFP responses for spatial rescue of dunce1. Brain activity in the 10 s immediately preceding a novelty effect (as shown in Figure 5). In this case, LFP activity for 3 frequency domains is in response to two identical squares (shown in the schema at the bottom of each column). The data follows exactly the outline of Figure 5. A significant effect was found for the c309 circuit in the 20–30 Hz range (P<0.05, Wilcoxon rank sum), indicating that in this particular strain flies responded to one square more than the other, immediately preceding a choice between a square and a cross. (3.00 MB TIF) [file pone.0005989.s005.tif]

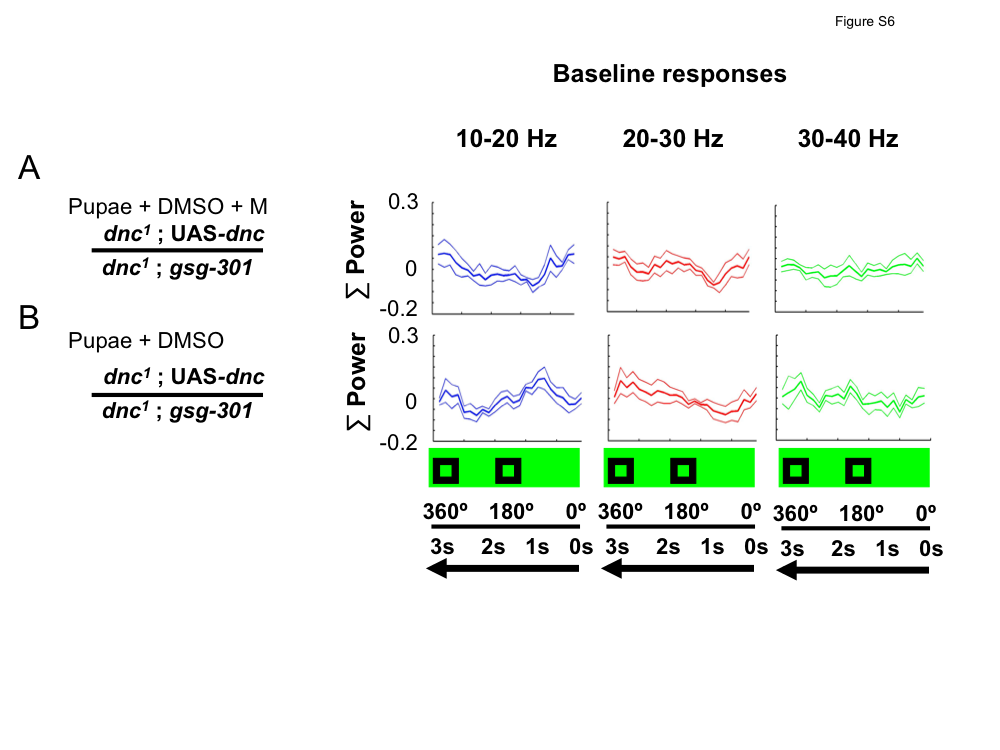

Supplement: Figure S6 — Baseline LFP responses for temporal rescue of dunce1. Brain activity in the 10 s immediately preceding a novelty effect (as shown in Figure 6). M = Mifepristone. No significant effects were detected. (3.00 MB TIF) [file pone.0005989.s006.tif]

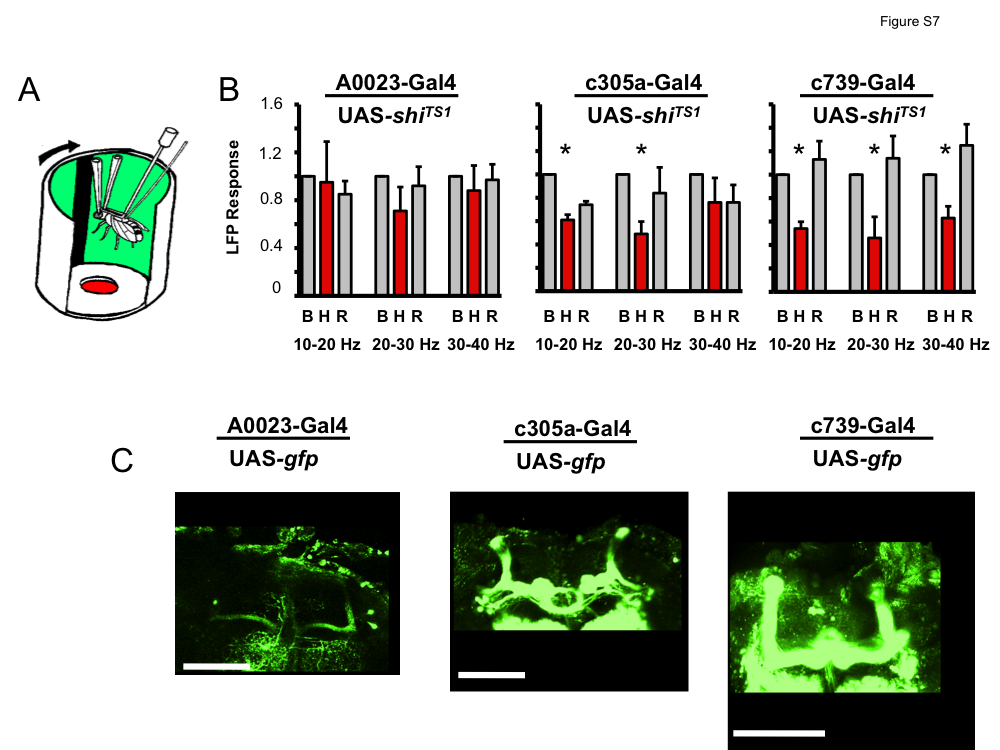

Supplement: Figure S7 — Electrophysiology of synaptic silencing in 3 mushroom body circuits. A. Recording arena setup. The visual stimulus is a moving dark bar on a lit green background. B. Brain responses to the visual for the Gal4 drivers A0023, c305a, and c739 [16] complemented by UAS-shibire. LFP responses are calculated as the normalized maximum - minimum [2] for three successive temperature conditions, for three frequency domains. B = baseline response, at 22°, H = response during heating at 38°C, R = recovery response at 22°. Calculations were for 100 s at each condition, averaged from a triplicate test per fly. N = 3 flies per genotype. * = significantly different from baseline, set at 1 (P<0.05, by t-test). C. UAS-GFP expression of the Gal4 lines tested in B. (3.00 MB TIF) [file pone.0005989.s007.tif]
